# Data of PCL-b-P(MMA-DMAEMA)_2_ characterization and related assays

**DOI:** 10.1016/j.dib.2017.09.010

**Published:** 2017-09-19

**Authors:** Camila Franco, Michelli Barcelos Antonow, Aline Beckenkamp, Andréia Buffon, Taise Ceolin, Marli Luiza Tebaldi, Gustavo Pozza Silveira, Silvia Stanisçuaski Guterres, Adriana Raffin Pohlmann

**Affiliations:** a Programa de Pós-Graduação em Ciências Farmacêuticas, Faculdade de Farmácia, Universidade Federal do Rio Grande do Sul, Av. Ipiranga, 2752, Porto Alegre 90610-000, RS, Brazil; ^b^Programa de Pós-Graduação em Nanotecnologia Farmacêutica, Faculdade de Farmácia, Universidade Federal do Rio Grande do Sul, Av. Ipiranga, 2752. Porto Alegre, 90610-000, RS, Brazil.; ^c^Departamento de Química Orgânica, Instituto de Química, Universidade Federal do Rio Grande do Sul, Av. Bento Gonçalves, 9500, Porto Alegre 91501-970, RS, Brazil

**Keywords:** Methacrylic copolymer, Polycaprolactone, Nanocapsules, pH-sensitive, Cell viability

## Abstract

The data presented here are related to the research paper entitled “PCL-*b*-P(MMA-*co*-DMAEMA)_2_ new triblock copolymer for novel pH-sensitive nanocapsules intended for drug delivery to tumors” by Franco et al. [1]. Characterization data of PCL-diol, macroinitiator Br-PCL-Br, homopolymers (PMMA and PDMAEMA) and copolymers (batch 1 and batch 2) analyzed by FTIR, SEC and NMR, as well as, characterization of PCL-NS formulation by laser diffraction and DLS analysis, initial nanocapsule formulations and 1**C**-NC and 2**C**-NC formulations, including hydrodynamic diameter at different pH media, and DMSO cytotoxicity.

**Specifications Table**

**Table:** 

Subject Area	*Chemistry, Biology, Pharmacy*
More specific subject area	*Copolymer synthesis and pH-sensitive nanocapsules*
Type of data	*Tables and figures*
How data was acquired	*FTIR spectrometer (Varian 640 FT-IR, USA), SEC by GPCMax tripledetector (Viscotek, Marvel Instruments Ltd, England, UK, columns of Styragel 10*^*4*^*, 10*^*5*^*, and 10*^*6*^*Å),*^*1*^*H NMR (300 MHz) and*^*13*^*C NMR (75 MH) by INOVA-300 (Varian, USA), laser diffraction (Malvern Mastersizer*^*®*^*2000, Malvern Instruments, UK), dynamic light scattering (DLS, Malvern Zetasizer instrument - NanoZS, Malvern Instruments, UK) and cytotoxicity in MCF-7 cells (ATCC*^*®*^*-HTB-22*^*TM*^*Rockville, MD, λ of 570 and 630 nm - SpectraMax M2, Molecular Devices)*
Data format	*Raw, analyzed*
Experimental factors	*Synthesis and products isolation by filtration and purification by impurities dissolution. Nanocapsules were analyzed as produced, without pre-treatment*
Experimental features	*Chemical characterization and identification of modifications induced by synthesis procedures or by formulation of materials*
Data source location	*Commercial reagent: PCL, MMA and DMAEMA*
Data accessibility	*Data is provided with this article*

**Value of data**

• Characterization spectra of the materials were compared with data from other works when developing a similar delivery system or copolymer synthesis.
• SEC and NMR data provided information on the efficiency of the copolymer synthesis and were useful for their identification.
• Nanocapsules parameters and it response to different pH media is innovative for scientific community since the copolymer maintains its integrity and expands upon acid pH.
• The bromide end-group of the copolymer permit application as active targeting system after covalent binding with ligands.

## 1 Data

**Fig. 1:**
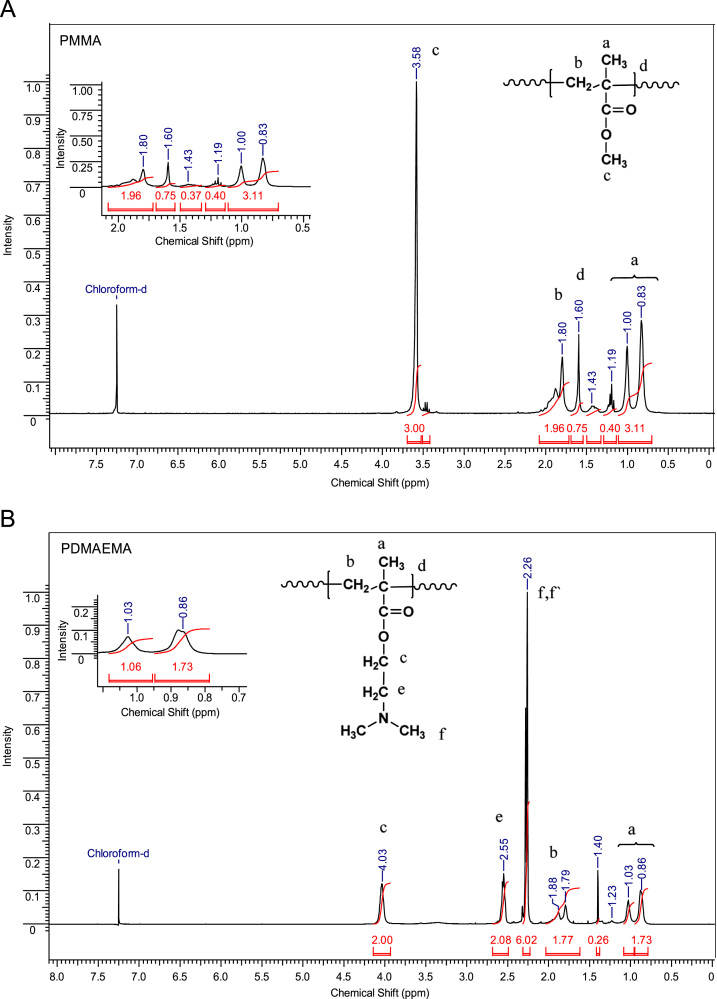
^1^H NMR (CDCl_3_, 300 MHz) spectra of PMMA (A) and PDMAEMA (B).

**Fig. 2:**
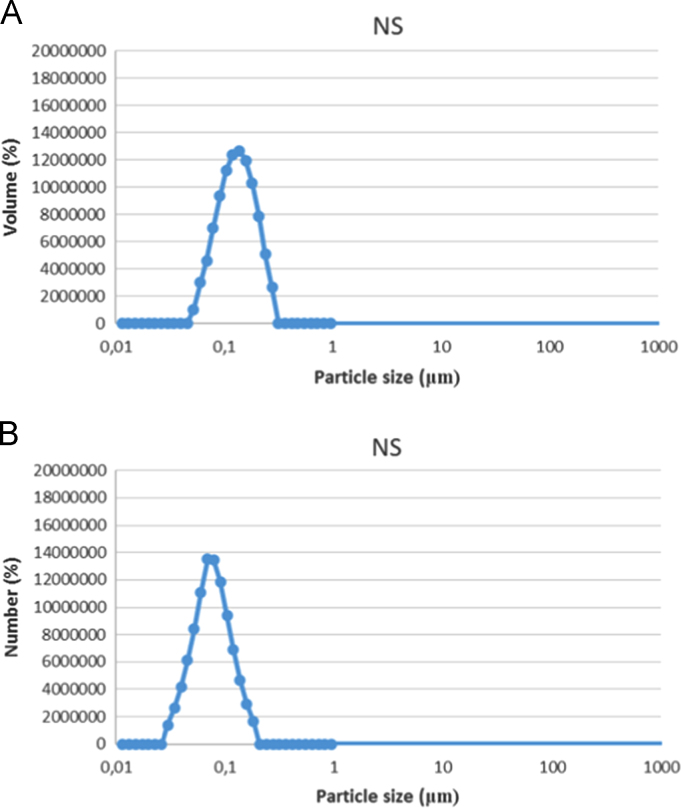
Size distribution profiles by laser diffraction analysis of PCL-nanospheres, expressed by volume (A) and by number (B) of particles.

**Fig. 3:**
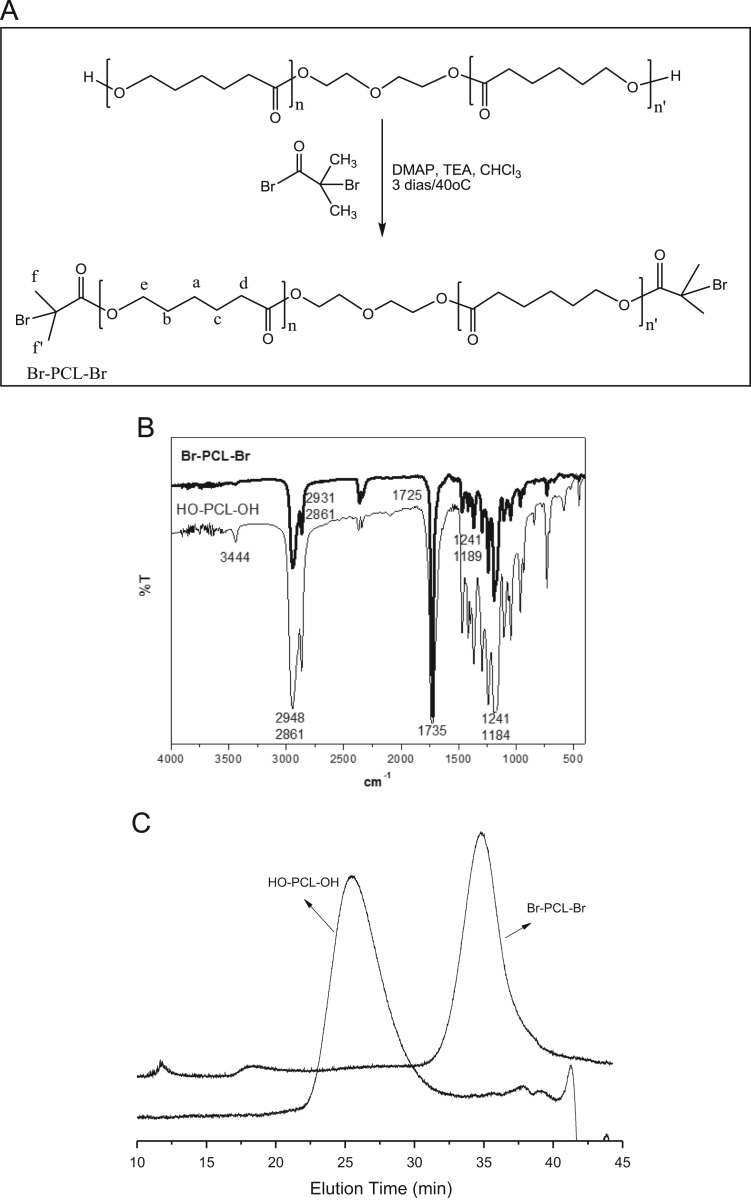
Macroinitiator Br-PCL-Br synthesis (A) and characterization of the product by FT-IR (B) and SEC (C).

**Fig. 4:**
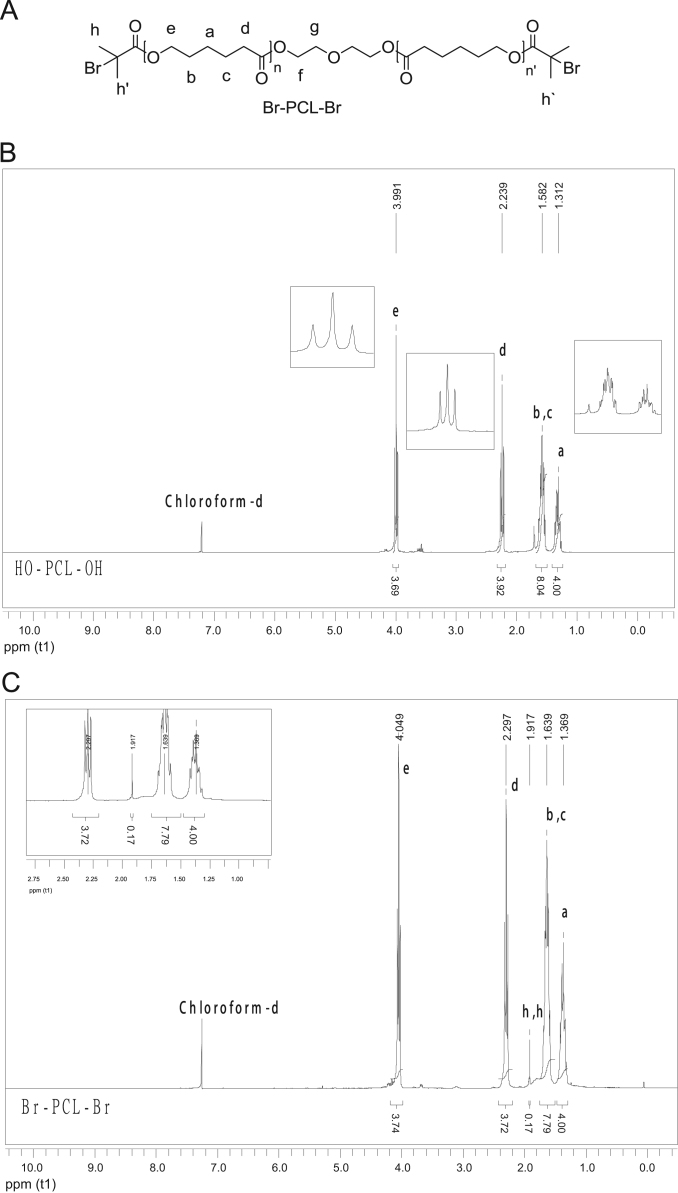
Macroinitiator Br-PCL-Br structure (A) and ^1^H NMR (CDCl_3_, 300 MHz, D) spectra of PCL-diol (B) and Br-PCL-Br (C).

**Fig. 5:**
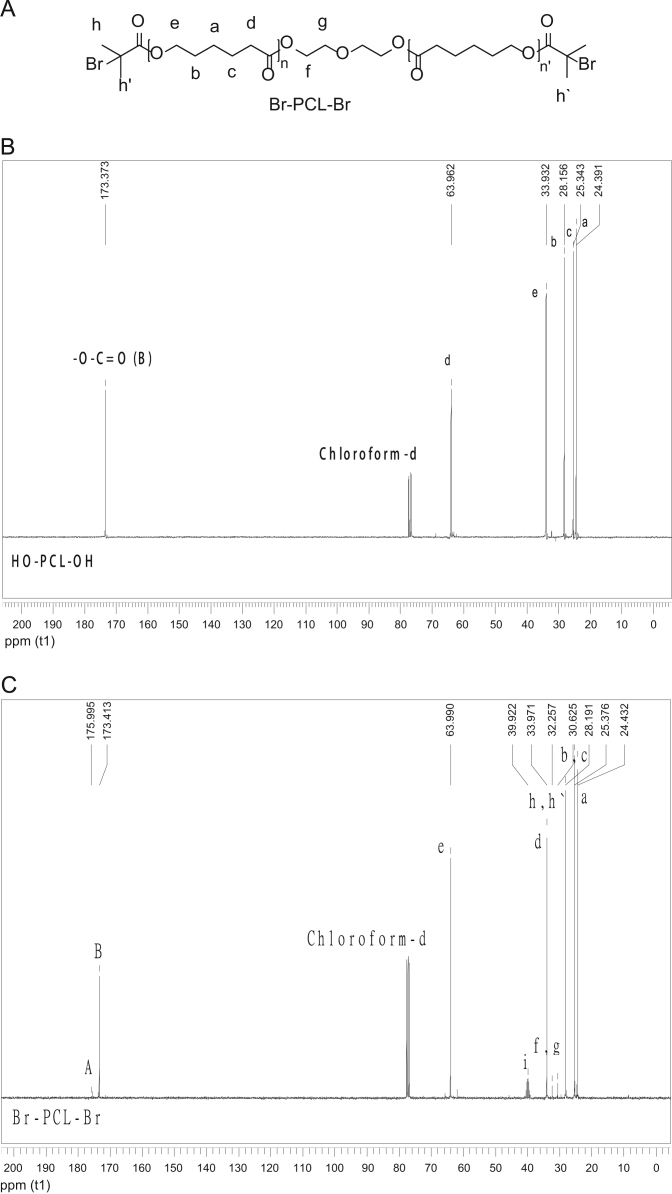
Macroinitiator Br-PCL-Br structure (A) and ^13^C NMR (CDCl_3_, 300 MHz) spectrum of PCL-diol (B) and Br-PCL-Br (C).

**Fig. 6:**
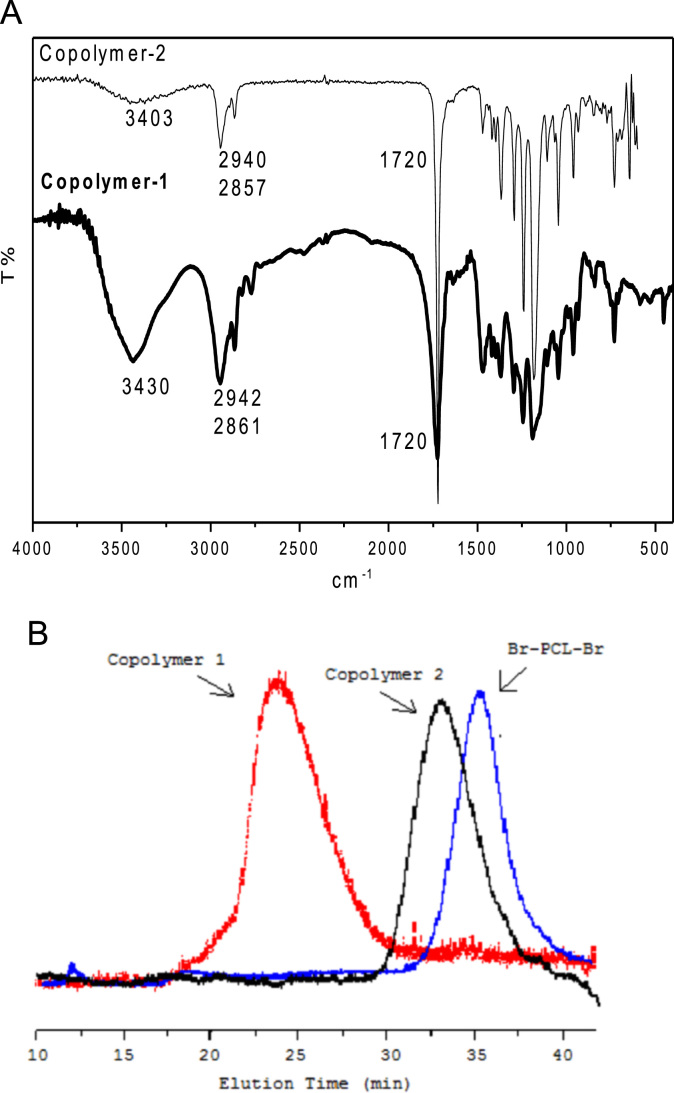
Characterization of PCL-*b*-P(DMAEMA-*co*-MMA)_2_ products (batches 1 and 2) by FT-IR (A) and SEC (B).

**Fig. 7:**
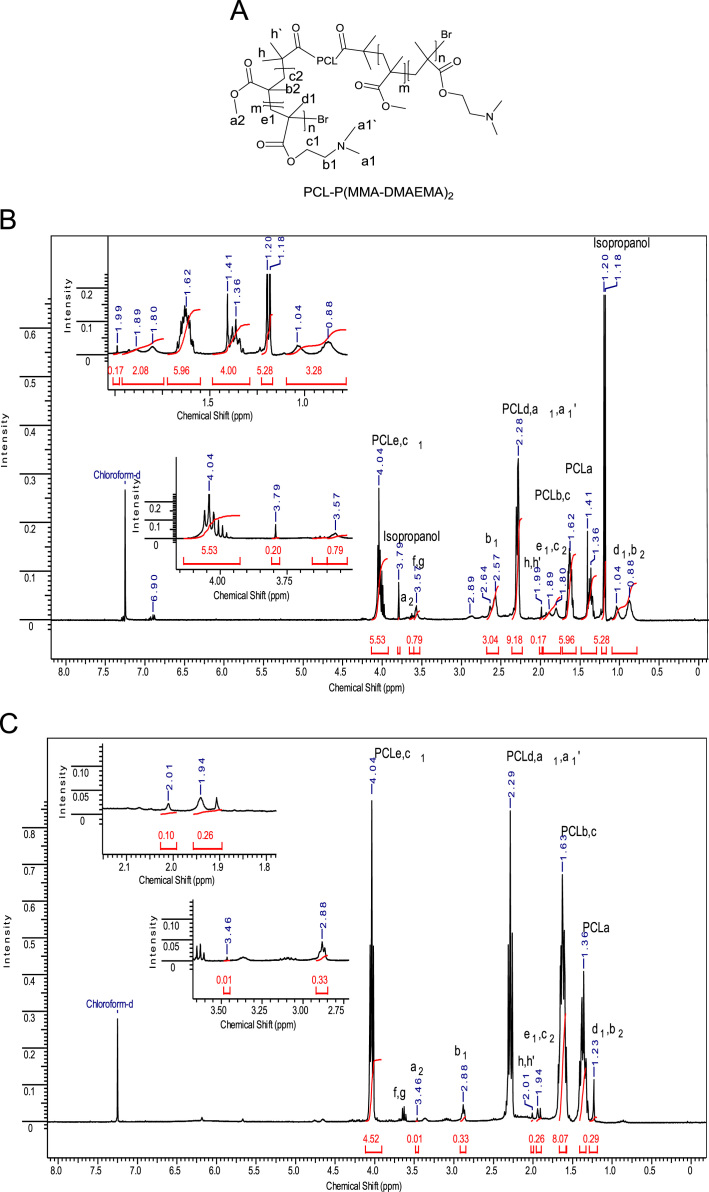
PCL-*b*-P(DMAEMA-*co*-MMA)_2_ structure (A) and ^1^H NMR (CDCl_3_, 300 MHz, D) spectra of PCL-*b*-P(DMAEMA-*co*-MMA)_2_ batch 1 (B) and batch 2 (C).

**Fig. 8:**
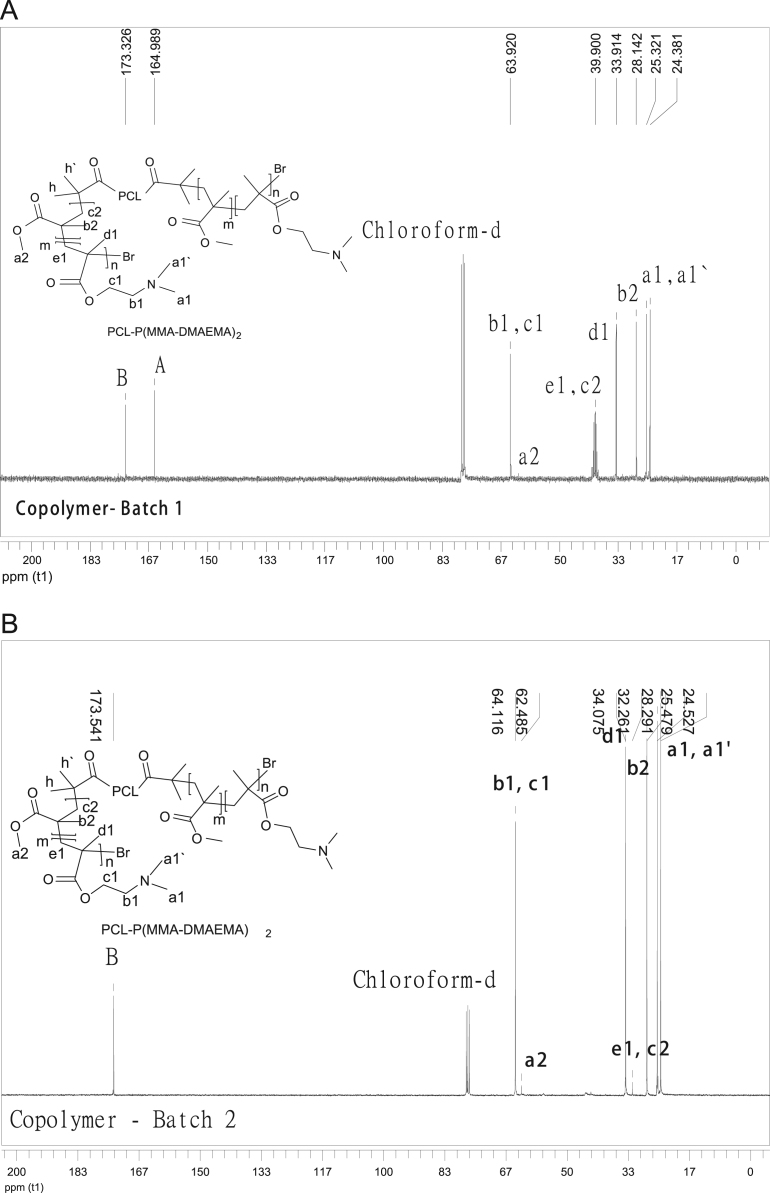
^13^C NMR (CDCl_3_, 300 MHz) spectra of PCL-*b*-P(DMAEMA-*co*-MMA)_2_ batch 1 (A) and batch 2 (B).

**Fig. 9:**
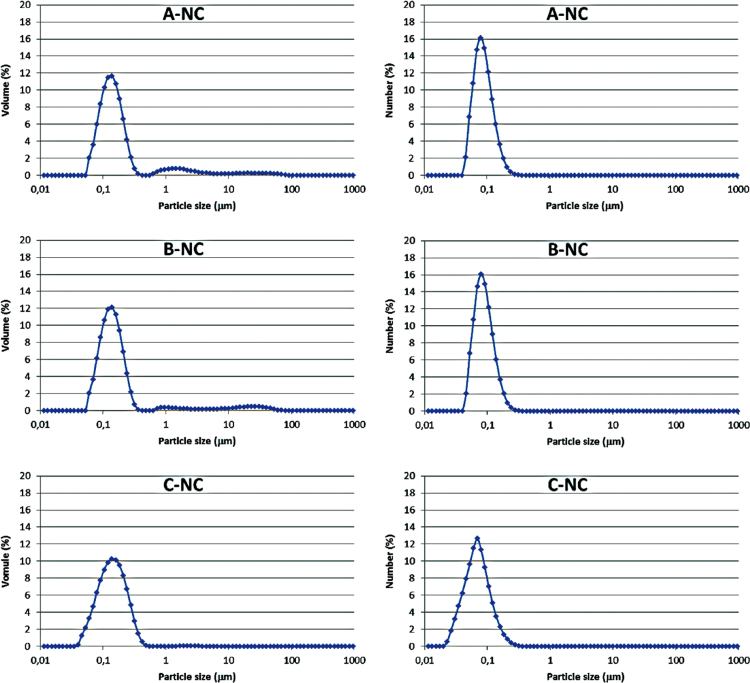
Size distribution profiles by laser diffraction of nanocapsule formulations: **A**-NC, **B**-NC and **C**-NC, expressed by volume of particles (left) and by number of particles (right).

**Fig. 10:**
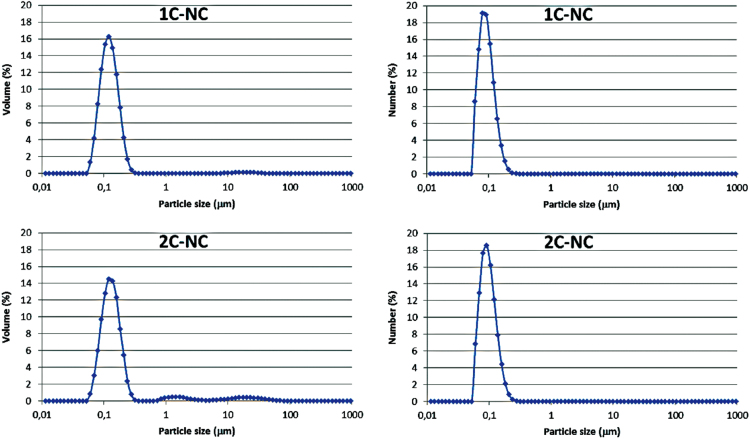
Size distribution profiles by laser diffraction of nanocapsule formulations: 1**C**-NC and 2**C**-NC, expressed by volume of particles (left) and by number of particles (right).

**Fig. 11:**
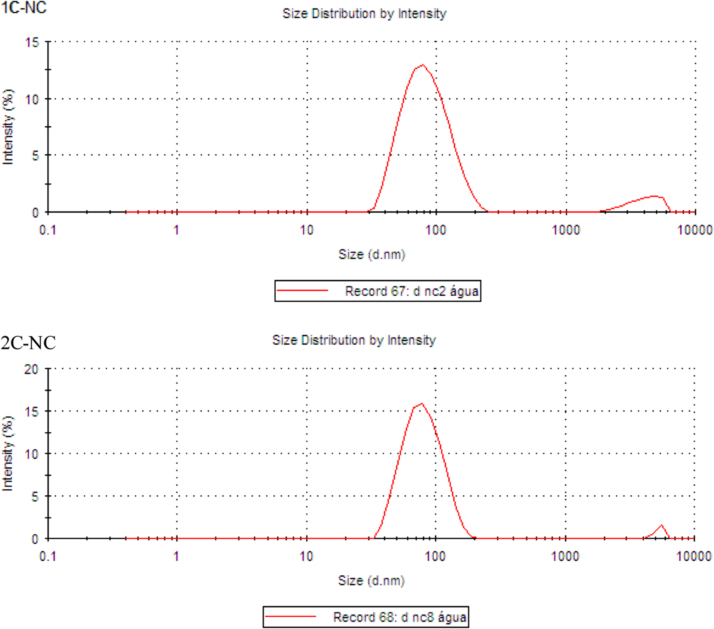
Size distribution profiles by DLS analysis of nanocapsule formulations: **1**C-NC and **2**C-NC, expressed by intensity (%).

**Fig. 12:**
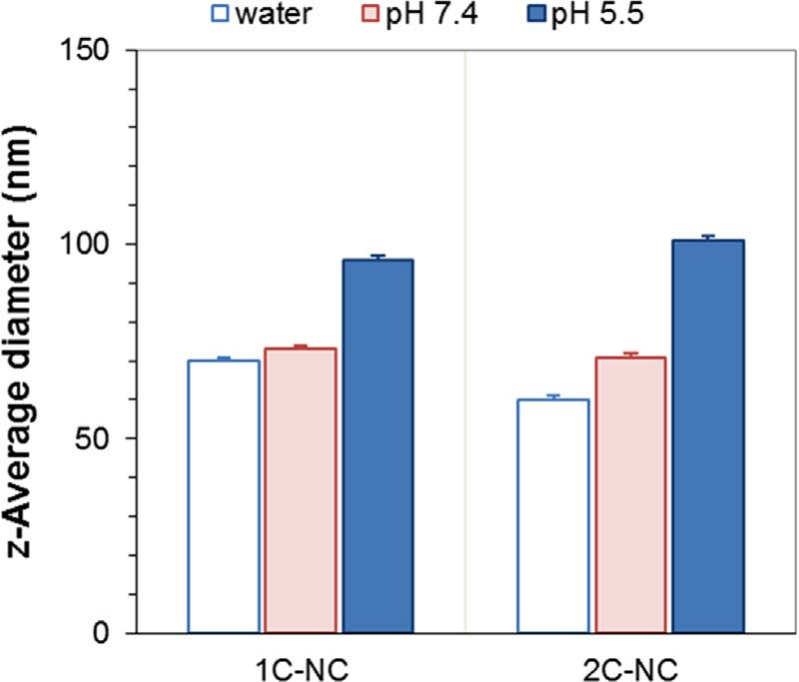
Hydrodynamic diameters (z-average) by dynamic light scattering of 1**C**-NC and 2**C**-NC in different media: ultrapure water, potassium phosphate buffer pH 7.4 and potassium phosphate buffer pH 5.5.

**Fig. 13:**
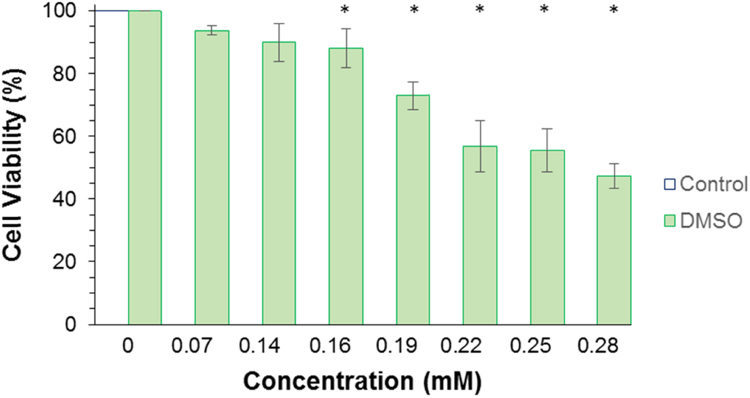
DMSO cytotoxicity assessed by MTT assay, after 24 hours of treatment in MCF-7 cells (n=1, quadriplicate). The culture medium was used as control. The symbol (*) represents the statistical diffferences between the sample and the control (ANOVA, F = 45.34, Fcritic = 2.57, p = 6.00.10^-11^ and HSD = 11.22).

The data presented in Section 1.1 is the ^1^H NMR analysis of the homopolymers PMMA and PDMAEMA (Fig. 1). Section 1.2 involves the profiles by laser diffraction of PCL-NS and its parameters (Fig. 2, Table 1). The data presented in Section 1.3 includes the synthesis of the macroinitiator and the characterization by FTIR and SEC analysis of the PCL-diol and Br-PCL-Br (Fig. 3), ^1^H NMR (Fig. 4) and ^13^C NMR (Fig. 5). Section 1.4 brings data referent to the copolymers (batch 1 and batch 2) with FTIR, SEC (Fig. 6), ^1^H NMR (Fig. 7) and ^13^C NMR (Fig. 8). The data contained in Section 1.5 is related to the characterization of nanocapsules formulations, as size distribution profiles of initial nanocapsule formulations (Fig. 9) and 1C-NC and 2C-NC formulations (Fig. 10), including its parameters (Table 2) and the DLS profile (Fig. 11) and it behavior in different pH (Fig. 12). Section 1.6 presented the DMSO cytotoxicity data (Fig. 13).

**Table 1:** Laser diffraction analysis of NS formulation.

Formulation	Diameters (μm)				Span_v_	Diameters (μm)				Span_n_
	D_4,3v_	d_0.1v_	d_0.5v_	d_0.9v_		D_4,3n_	d_0.1n_	d_0.5n_	d_0.9n_	
PCL-NS	0.130	0.071	0.122	0.203	1.077	0.130	0.041	0.071	0.122	1.142

**Table 2:** Diameters and polydispersity (Span) determined by laser diffraction analysis of formulations 1**C**-NC e 2**C**-NC.

Formulation	Diameters (μm)				Span_v_	Diameters (μm)				Span_n_
	D_4,3v_	d_0.1v_	d_0.5v_	d_0.9v_		D_4,3n_	d_0.1n_	d_0.5v_	d_0.9v_	
1**C**-NC	0.600± 0.49	0.103±0.00	0.120±0.01	0.174±0.09	0.781±0.64	0.125±0.06	0.061±0.00	0.085±0.00	0.131±0.00	0.795±0.04
2**C**-NC	0.794±0.53	0.08±0.00	0.134±0.02	0.318±0.18	1.682± 0.95	0.143± 0.03	0.053±0.01	0.079±0.02	0.127±0.00	0.973±0.33

### 1.1 ^1^H NMR spectra of homopolymers PMMA and PDMAEMA

### 1.2 Laser diffraction profiles expressed by volume and number of particles

### 1.3 Macroinitiator synthesis and characterization

See Fig. 3, Fig. 4, Fig. 5.

### 1.4 Copolymers characterization

See Fig. 6, Fig. 7, Fig. 8.

### 1.5 Characterization of nanocapsules formulations

See Fig. 9, Fig. 10, Fig. 11, Fig. 12 and Table 2.

### 1.6 DMSO cytotoxicity

## 2 Experimental design, materials and methods

The methodologies to obtain the data exposed here are described in [1] and in cited references.

PCL-NS was prepared using PCL (14,000 g mol^-1^, 0.0301 g) solubilized in 30 mL of acetone:ethanol 1:1 (v/v) and injected into an aqueous dispersion (60 mL) of polysorbate 80 (0.0518 g), having its volume reduced to 10 mL.

Copolymers synthesis was performed with Br-PCL-Br macroinitiator (1.75 g, 0.14 mmol), DMAEMA (1 g, 6.36 mmol), MMA [65 mg, 0.65 mmol for batch **1** or 19 mg, 0.19 mmol (1 drop) for batch **2**] in 2 mL of anisole were added in a bottle flask and stirred under argon at room temperature for 15 min. Then, a mixture of 0.1 mL of (CuBr (I) (6 mg, 0.04 mmol), PMDETA (140 mg, 0.81 mmol) in 1 mL of anisole) was added by a syringe at once. After 2 min, a solution of Tin (II) 2-ethylhexanoate (32 mg, 0.08 mmol) in anisole (2 mL) was drop-wised using a syringe. Then, the temperature was raised to 90 °C and the reaction was maintained under stirring for 24 hours. After cooling to room temperature, THF (5 mL) was added and the copolymer was precipitated in cold cyclohexane (50 mL). To remove the catalyst and non-reacted monomers, the crude solid was dissolved in 2-propanol (5 mL) and precipitated in cold cyclohexane (50 mL). The precipitate was isolated by filtration and dried under vacuum (Edwards Weg ® C56 0698, Brazil).

Initial nanocapsule formulations and 1C-NC and 2C-NC formulations are described in the Table 3.

**Table 3:** Nanocapsules initial formulations and formulations.

Materials and quantities^a^		**Initial formulation**		**Formulations**	
	**A**-NC	**B**-NC	**C**-NC	**1C-NC**	**2C-NC**
PCL-*b*-P(MMA-*co*-DMAEMA)_2_ Batch **1** (g)	0.0103	0.0103	0.0320	0.0341	0
PCL-*b*-P(MMA-*co*-DMAEMA)_2_ Batch **2** (g)	0	0	0	0	0.0304
Sorbitan monostearate (g)	0.0042	0.0042	0	0	0
CCT (oil) (g)	0.0163	0.0163	0.0540	0.0574	0.0521
Acetone (mL)	25	25	15	15	15
Ethanol (mL)	0	0	15	15	15
Polysorbate 80 (g)	0.0084	0.0008	0.0520	0.0538	0.0514
Water (mL)	53	53	53	60	60

a Final volume after evaporation = 10 mL
